# Religiousness and health-related quality of life of older adults

**DOI:** 10.1590/S0034-8910.201504900541

**Published:** 2015-09-10

**Authors:** Gina Andrade Abdala, Miako Kimura, Yeda Aparecida de Oliveira Duarte, Maria Lúcia Lebrão, Bernardo dos Santos

**Affiliations:** I Programa de Pós-Graduação em Promoção da Saúde. Centro Universitário Adventista de São Paulo. São Paulo, SP, Brasil; II Escola de Enfermagem. Universidade de São Paulo. São Paulo, SP, Brasil; III Faculdade de Saúde Pública. Universidade de São Paulo. São Paulo, SP, Brasil

**Keywords:** Aged, Health Status, Religion and Psychology, Spirituality, Interpersonal Relations, Quality of Life, Socioeconomic Factors, Health Surveys

## Abstract

**OBJECTIVE:**

To examine whether religiousness mediates the relationship between sociodemographic factors, multimorbidity and health-related quality of life of older adults.

**METHODS:**

This population-based cross-sectional study is part of the Survey on Health, Well-Being, and Aging (SABE). The sample was composed by 911 older adults from Sao Paulo, SP, Southeastern Brazil. Structural equation modeling was performed to assess the mediator effect of religiousness on the relationship between selected variables and health-related quality of life of older adults, with models for men and women. The independent variables were: age, education, family functioning and multimorbidity. The outcome variable was health-related quality of life of older adults, measured by SF-12 (physical and mental components). The mediator variables were organizational, non-organizational and intrinsic religiousness. Cronbach’s alpha values were: physical component = 0.85; mental component = 0.80; intrinsic religiousness = 0.89 and family APGAR (Adaptability, Partnership, Growth, Affection, and Resolve) = 0.91.

**RESULTS:**

Higher levels of organizational and intrinsic religiousness were associated with better physical and mental components. Higher education, better family functioning and fewer diseases contributed directly to improved performance in physical and mental components, regardless of religiousness. For women, organizational religiousness mediated the relationship between age and physical (β = 2.401, p < 0.01) and mental (β = 1.663, p < 0.01) components. For men, intrinsic religiousness mediated the relationship between education and mental component (β = 7.158, p < 0.01).

**CONCLUSIONS:**

Organizational and intrinsic religiousness had a beneficial effect on the relationship between age, education and health-related quality of life of these older adults.

## INTRODUCTION

The Brazilian population has been aging steadily and sharply. The percentage of older adults in the city of Sao Paulo was 12.5% in 2012, slightly larger than in the state of Sao Paulo (12.1%) and Brazil (11.3%).^6^ In 2050, older adults will exceed 22.7% of the total population.^a^

Older adults commonly suffer from chronic non-communicable diseases. Physical, emotional and psychosocial changes resulting from these diseases may compromise, to varying degrees, their perception of well-being and quality of life (QOL). In addition, multimorbidity can interfere with the performance of instrumental activities, being a predictive factor for worse health-related quality of life (HRQOL) in the elderly.^11^

Other factors that may also explain the HRQOL of seniors are age, sex, education and family functioning (family APGAR – Adaptability, Partnership, Growth, Affection, and Resolve). The higher the age, the worse the performance in HRQOL dimensions.^b^ Women present worst levels in both the physical and mental components.^20^ The more years of schooling, the better the physical and mental HRQOL.^6^ ^,^ ^21^ Good family functioning is also predictive of better HRQOL.^2^

Population ageing raises disease burdens and disabilities. However, preventing these diseases is effective even in old age; therefore, it is the main focus to change the current profile of chronic diseases.^23^

In the Survey on Health, Well-Being, and Aging (SABE), from 2000,^c^ there was inequality in the use and access to health services and inadequacy in the older adults care model, indicating a need for public policies that consider this population’s particularities.^13^

When planning actions aimed at older adults, it is necessary to know this population’s health conditions and quality of life as well as their determining factors. Quality of life is a multidimensional and subjective concept. In the absence of conceptual consensus, terms such as satisfaction with life, well-being, happiness, good life, valuing life and functional state have been used.^9^ HRQOL is defined as the value given to the duration of life, modified by damage, functional states, health harms, treatments or public policies, perceptions and social opportunities influenced by the disease. HRQOL was introduced to narrow the focus on the effects of health issues and therapeutic interventions on quality of life.^16^ Several studies on aging, health and quality of life assess the protective effect of religious/spiritual practices and beliefs. A growing number of studies has shown positive correlation between religion/spirituality and physical and mental health.^3^

The importance of religion and its practice related to QOL was positively evaluated by 50 individuals aged 65 to 86 years.^18^ Religion was considered extremely important for their lives (p ≤ 0.05), giving strength to withstand problems, losses and struggles. In addition, contact with the divine is important because it brings security and spiritual comfort.^18^

Religiousness is understood as the degree to which the individual believes in, follows and practices a religion. It includes the organizational, non-organizational and intrinsic dimensions. The organizational dimension is related to public participation in religious services in temples or churches. The non-organizational dimension involves practicing activities outside of a religious institution. The intrinsic dimension refers to beliefs, psychological aspects of religion, knowledge and attitudes related to the religious experience.^11^

Religion can positively impact physical and mental health through a social support network, reduction of unhealthy behaviors, decrease in blood pressure and muscle tension during prayer and meditation and greater adherence to medical treatment and preventative care.^1^

Religiousness is a striking feature of the Brazilian population: 95.0% of the population have a religion, 83.0% consider it very important in their lives and 37.0% attend a religious service at least once a week.^19^

In a comparative study on the religiousness profile of older adults in Sao Paulo who participated in SABE in 2000 and 2006, it was observed that having a religion and giving it importance positively impacted the perception of health.^d^

Another investigation, based on the same study, analyzed the factors associated with HRQOL. Among several potential predictors, two religious variables were included (religious affiliation and importance of religion), but none of them were associated with the physical and mental components of Short Form-12 (SF-12).^b^

The aim of this study was to examine whether religiousness mediates the relationship between sociodemographic factors, multimorbidity and health-related quality of life of older adults.

## METHODS

It was an observational, cross-sectional study, with the quantitative approach of SABE. The sample was composed of 911 individuals aged 60 years or over in the urban area of the city of Sao Paulo, SP, Southeastern Brazil. Those with cognitive decline and whose questionnaires were not answered were excluded from the sample.

The independent variables were age, sex, education, number of diseases and family functioning (family APGAR). Family APGAR ranges from zero (the best) to 20 (the worst).

The dependent variables were the dimensions of the physical and mental components of HRQOL as assessed by SF-12. The mediator variables were the organizational, non-organizational and intrinsic dimensions of religiousness.

Data were analyzed by structural equation modeling (SEM), using the Statistical Package for the Social Sciences (AMOS-SPSS, version 22). SEM is a multivariate technique that allows simultaneously analyzing a series of relationships between measured variables and latent constructs.^7^ For the indirect effect of a variable and the outcome in the components, the values obtained are multiplied.^7^

We used the maximum likelihood method, commonly used to estimate parameters in structural equation models. A 90% confidence interval (CI) was used. The fit indexes used were the root mean square error of approximation (RMSEA), the comparative fit index (CFI) and the Tucker-Lewis index (TLI).^7^

To measure questionnaire reliability, Cronbach’s alpha was estimated. For the physical component (PC) of HRQOL, the coefficient was 0.85; for the mental component (MC), 0.80; for the intrinsic religiousness (IR), 0.89; and for the family APGAR, 0.91.

Student’s t-test was also conducted to compare the means of the physical and mental components by sex.

This study was approved by the Ethics and Research Committee of Faculdade de Saúde Pública, Universidade de São Paulo, and by the *Conselho Nacional de Ética em Pesquisa* (CONEP – National Committee for Ethics in Research, Process 1345, 3/14/2006).

## RESULTS

The population sample showed higher percentage of women (58.9%). Age ranged from 60 to 94 years, with a mean of 71.8 (SD = 8.0) for women and 72.3 (SD = 9.0) for men. Men prevailed only in the age group of 60 to 69 years, while women were the majority in the age groups of 70 to 79 years, and 80 years and over (p < 0.001).

As for education, women showed a mean of 3.8 years of schooling (SD = 3.5) and men, 4.5 (SD = 4.2). There were more illiterate women (18.9%) than men (11.7%) (p < 0.001).

Family APGAR also varied by sex: women had a mean of 2.4 (DP = 4.6) and men, 2.2 (SD = 4.0). Regarding family functioning, men and women presented 90.6% and 89.2%, respectively (p < 0.001).

The mean number of diseases mentioned by women and by men was 1.6 (SD = 1.2) and 1.3 (DP = 1.1), respectively. The most cited diseases among older adults were: hypertension (64.7% of women and 57.3% of men), diabetes (with a prevalence of 21.6% in women and 19.5% in men) and osteoarticular diseases (41.6% of women and 18.7% of men).

As to religion, Catholic was the most predominant one (66.2%), followed by Evangelical (22.3%) and Spiritualist (5.4%). A lower percentage claimed to be Protestant (1.0%), Buddhist (0.6%) or not having any religion (1.7%). By associating sex and religious denomination, we found that there were more Catholic men (72.9%) than women (61.2%) and, consequently, more Evangelical women (26.3%) than men (16.6%) (p < 0.001).

As for the means of the physical and mental components of the HRQOL according to sex, men showed higher means for both components. The mean of the physical component was 47.6 (DP = 7.9) for men against 43.4 (DP = 10.3) for women; for the mental component, it was 55.0 (DP = 7.8) for men against 53.7 (DP = 9.1) for women.

Table shows the data relating to religiousness dimensions. Older women attend more religious cults, practice more private religious activities and give more importance to religion (p < 0.001). They also consider that “religion gives strength to face and help understand the hardships of life” the most. In addition, for them, religion brings more “meaning to life”. They also consider themselves more religious than men (p < 0.001).

**Table:** Distribution of older adults according to sex and religiousness dimensions. Sao Paulo, SP, Southeastern Brazil, 2006. (N = 911)

Religiousness dimensions	Female	Male	Total

	%	%	%
ORA - Frequency of church attendance			
Never	7.4	12.5	9.5
Several times a year	20.1	36.4	26.8
Once or twice a month	15.7	12.0	14.2
Almost every week	25.1	18.6	22.4
More than once a week	31.7	20.4	27.1
NORA - Frequency of praying or religion practicing			
Hardly ever or never	2.2	6.9	4.1
Only on special occasions	0.7	9.7	4.4
Several times a week	3.8	7.1	5.1
Once a day	35.9	41.9	38.4
Several times a day	57.5	34.4	48.0
IR - Importance of religion in life			
Not important	1.7	5.0	3.1
Moderate	3.0	6.7	4.5
Important	95.2	88.3	92.4
IR - To what extent religion gives strength to face hardships			
None	1.7	6.6	3.7
Not much	4.3	12.8	7.8
Very much	50.9	51.0	51.0
Completely	43.1	29.6	37.5
IR - To what extent religion helps understand hardships			
None	2.1	8.3	4.7
Not much	7.2	12.3	9.3
Very much	50.0	52.5	51.0
Completely	40.7	26.9	35.0
IR - Religion gives meaning to life			
Never	2.2	8.8	5.0
Sometimes	6.9	12.2	9.1
Very much	42.0	47.9	44.4
Totally	48.9	31.0	41.5
IR - How religious individuals consider themselves			
None	2.6	5.1	3.6
Not much	6.0	11.0	8.1
A little	27.9	40.9	33.2
Very much	63.5	43.1	55.1

Source: SABE study.^c^

ORA: organizational religious affiliation; NORA: non-organizational religious affiliation; IR: intrinsic religiousness

**Figure 1:**
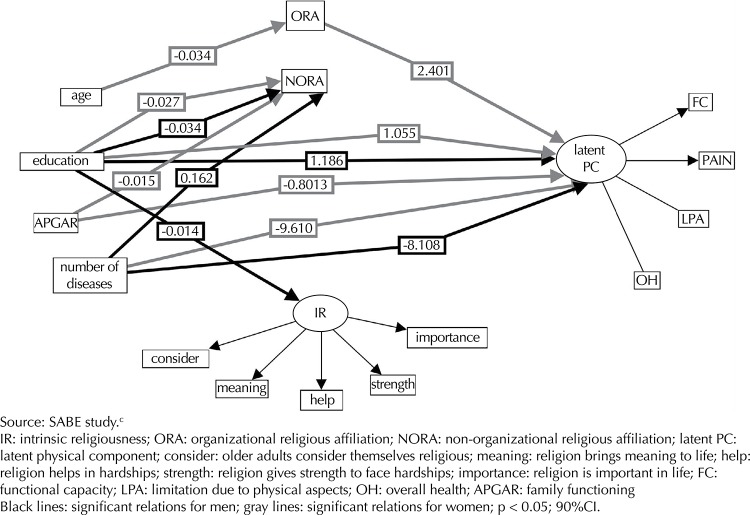
Representation of the structural and measurement model using structural equation modeling with the influences of the variables on the physical component for older men and women. Sao Paulo, SP, Southeastern Brazil, 2006.

Applying structural equation modeling, age influenced organizational religiousness (β = -0.034, p < 0.01), and the latter influenced the physical component of women (β = 2.401, p < 0.01). The estimation of the model was TLI = 0.613; CFI = 0.731; RMSEA = 0.08 (p ≤ 0.05, 90%CI) (Figure 1).

Religiousness was not a mediator between sociodemographic factors, diseases and physical component in men. However, higher education (β = 1.186, p = 0.04) and fewer diseases (β = 8.108, p < 0.01) directly improved the performance in the physical component of men, regardless of religiousness, showing that those variables directly influence health-related quality of life in older persons.

The variables that improved the physical component of women, regardless of the religiousness dimensions, were: higher educational level (β = 1.055, p = 0.04), better family functioning (β = 8.03, p < 0.01) and fewer diseases (β = 9.61, p < 0.01).

**Figure 2:**
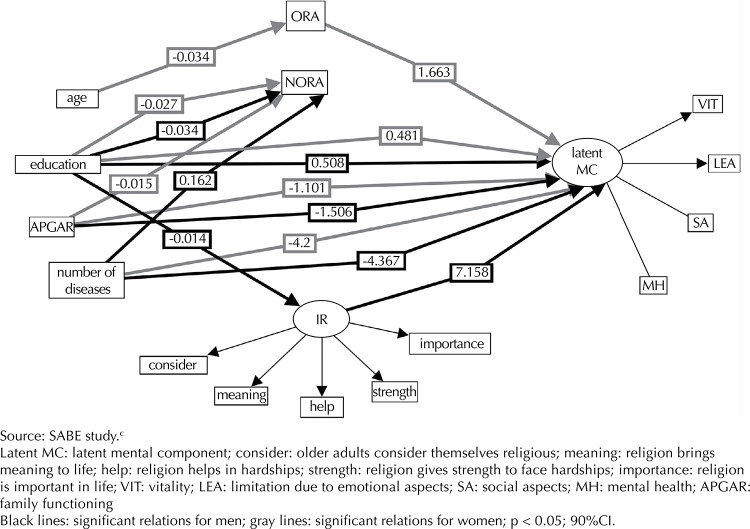
Representation of the structural and measurement model using structural equation modeling with the influences of the variables on the mental component for older men and women. Sao Paulo, SP, Southeastern Brazil, 2006.

Similar to the physical component, age was also associated with women’s organizational religiousness (β = -0.034, p < 0.01), which was associated to the mental component of these women (β = 1.661, p < 0.01). The estimation model was TLI = 0.613; CFI = 0.731; RMSEA = 0.08 (p ≤ 0.05, 90%CI) (Figure 2). The total indirect effect between age, religiousness and the physical component of older women was: age → organizational religiousness → physical component = -0.034 × 2.401 = -0.0813 (p ≤ 0.05), i.e., organizational religiousness (going to church) decreased 0.034 for each extra year of age; however, it increased the performance in the physical component of those older adults’ HRQOL by 2.401.

**Figure 3:**
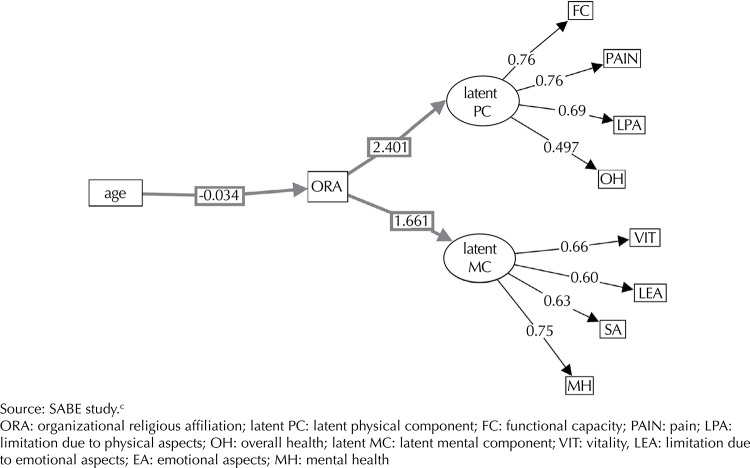
Synthesis of structural equation modeling for the physical and mental components, women. Sao Paulo, SP, Southeastern Brazil, 2006.

Analyzing the total indirect effect of the relationship between age, organizational religiousness and mental component of the older women, we obtained the following result: age → organizational religiousness → MC = -0.034 × 1.663 = -0.565. We can infer that the indirect effect of age on the mental component, mediated by organizational religiousness in women, was -0.564. The mental component dimensions of higher loads were mental health (0.75) and vitality (0.66) (Figure 3).

**Figure 4:**
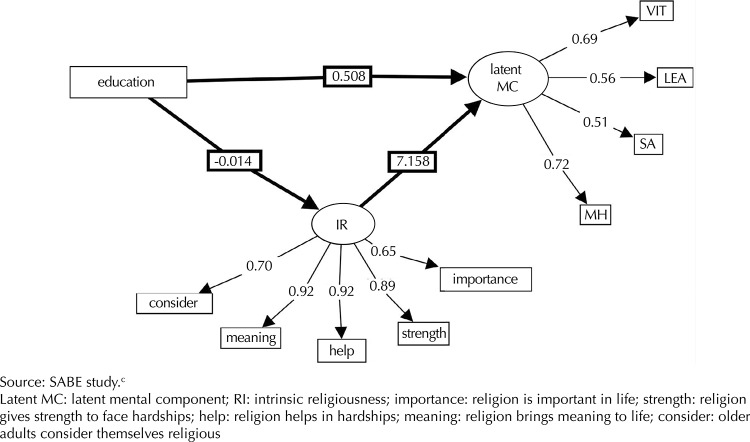
Synthesis of structural equation modeling for the mental component, men. Sao Paulo, SP, Southeastern Brazil, 2006.

Observing the simple model between education and the mental component of men, mediated by intrinsic religiousness, we found the following total effect (direct + indirect): Education → intrinsic religiousness → mental component (-0.014 × 7.158) + (0.507) = -0.1002 + 0.507 = 0.4067 (Figure 4).

## DISCUSSION

In this study, it was possible to observe that older women are more religious than older men in all religiousness dimensions, especially in church attendance. Despite the cultural differences of each country and the religious upbringings of each person, the literature usually indicates a difference between the sexes, showing that women are more open to express their religious feelings, comment, participate and engage in church tasks. They are also more intimate with God at the moment of death, while men are more passive and share less feelings.^20^

Even though their religiousness levels were higher than men’s, older women showed worse scores on the physical and mental components of HRQOL. In a study with 1,942 older women from California, USA^22^ that used SF-36, a similar physical component score was found (43.4; SD = 11.3), while the mental component was slightly higher (56.6; SD = 7.3). Women also live longer with physical disabilities and worse quality of life in the physical domain, especially the less educated ones.^21^

Testing the model of how sociodemographic and multimorbidity variables affect the HRQOL of older people, with religiousness as a mediator, we observed that age was a negative predictor for the physical component of the HRQOL of older women. However, organizational religiousness acted as a mediator variable in this relationship, predicting a better quality of life in their physical component. In this population, 49.5% attend religious cults almost every week, once or more than once a week, the majority being female.

Church attendance promotes social support and is a regular activity for many older people.^6^ In a study with 426 older adults of a community in Canada, spirituality was not a significant factor to improve quality of life (r = 0.19). However, the strongest predictors for quality of life were social support (*t* = 9.87, p < 0.001) and satisfaction about one’s own health (*t* = 6.854, p < 0.001), influenced by the social life that comes from being part of a religious community.

In the present study, religiousness only presented a mediator relationship with the physical component for women, possibly because they attend religious cults more frequently, which creates a social support network and helps them cope with adverse diseases.^6^

Higher education and fewer diseases acted as direct predictors of a better physical component of HRQOL in older adults of both sexes. These data corroborate the results of other studies.^6^ ^,^ ^11^ ^,^ ^21^

Analyzing the same model in relation to the HRQOL mental component, we observed that age also acted as a negative predictor for that component in older women. Nonetheless, organizational religiousness acted as a mediator variable in this relationship, predicting better mental health

A study with 499 older people from North Carolina, USA investigated public religious involvement social factors of quality of life, concluding that more deeply religious individuals were more likely to see friends (62.0%), had better self-rated health (51.0%), fewer depressive feelings (63.0%) and found life more exciting (49.0%) than the less religious. The study also showed that those with physical disabilities benefited more from religiousness, both subjective and related to public practice.^8^

Belonging to a religion and valuing it is an important support mechanism for older people to face their daily problems, contributing to greater satisfaction with life and less feeling of helplessness and hopelessness.^5^

Other studies with older adults confirm that the greater the intrinsic religious involvement, the greater the satisfaction with life.^3^ ^,^ ^4^ Examining the interaction between religion and spirituality in 277 older people from a cohort in Kansas City, USA, the authors found no relationship between religion and HRQOL, but spirituality and quality of life were associated (p < 0.01) with self-reported health status/overall health, even after adjusting for covariates age, race, education and depression. Daaleman et al^4^ reported that spirituality is an explanatory factor for the subjective health status in older people.

In the present study, schooling was inversely related to intrinsic religiousness and the latter was positively related to the mental component of men, i.e., intrinsic religiousness improved the relationship between schooling and their mental component.

The variable education (greater number of years in school) also improved directly the performance of the mental component of older adults of both sexes in this study. In a study with 82 older people from a community in Campinas, SP, Southeastern Brazil, higher levels of education and income were associated with better QOL.^6^

Other factors that also affected the mental component directly in this study were family APGAR (less family dysfunction) and fewer diseases.

The role of the family is being considered essential to predict better quality of life in older adults. In a study with 210 older people in Portugal, quality of life was higher among those with better family functioning, which should be considered when planning actions to promote quality of life for older adults.^2^

Multimorbidity, which may interfere with going to church due to difficulty in doing so by themselves, was also a predictor for worse HRQOL. It acts as a HRQOL suppressor variable, hampering its good performance.^11^

The design of this study did not allow establishing a causal relationship, but a predictor *versus* consequence one, in which some sociodemographic variables predict better HRQOL.

The scarce scientific literature on the relationship of multimorbidity and sociodemographic variables with HRQOL, mediated by religiousness and especially using SEM to analyze data, undermines the approximation of results for comparison purposes.

HRQOL-related factors of older adults draw attention to possible predictors, which will help unveil the best path in geriatric physical and mental health care, suggesting more research in this area.

Although religious or health professionals may diverge on the benefits or risks for the most serious health problems, people struggling with difficult situations such as multimorbidity, losses and stress can use their belief and religious practices to readapt, since they are one of the most important factors to endure stress.^12^

There are at least 20 scales that help diagnose and direct spiritual care to the individual. Eighteen of them were validated in Brazil and the authors suggest using them for diagnosing with the purpose of providing nursing care in a holistic perspective.^15^

Another spirituality scale recently adapted and psychometrically validated in Brazil is the Daily Spiritual Experience Scale, applied to 179 medical and surgical patients, showing reliability and validity for hospitalized patients.^10^

SEM statistical analysis was useful to clarify the goals proposed and observe the direct and indirect effects of independent variables in relation to older adults’ HRQOL, mediated by religiousness.

This analysis helped fill the gaps of the other studies found. Therefore, the hypotheses were proven, statistically showing that individuals of older age, lower education, better family functioning and fewer chronic diseases will have a higher level of religiousness. It was also proved that higher levels of organizational and intrinsic religiousness are predictors for better physical and mental quality of life in older adults.

Considering what we found in the literature about the importance of comprehensive care to older people and the important findings of this study, we recommend that health professionals be prepared for this; that they have communication and intervention skills in the field of religion and spirituality, because religiousness can be used as increased care for this demographic, increasing their health-related quality of life.

With population aging, it is necessary to promote health by decreasing the number of coexisting diseases, increasing family functioning, encouraging the increase in the educational level and promoting the mediator effect of religiousness on the improvement in HRQOL.

## Footnotes

a Instituto Brasileiro de Geografia e Estatística. Comunicação Social 27 de Novembro de 2008. Brasília (DF); 2008 [cited 2012 Jan 26]. Available from: http://www.ibge.gov.br/home/presidencia/noticias/noticia_visualiza.php?id_noticia=1272&id_pagina

b Ribeiro KT. Fatores associados à qualidade de vida relacionada à saúde de idosos residentes no município de São Paulo – Estudo SABE: Saúde, Bem-Estar e Envelhecimento [thesis]. São Paulo (SP): Faculdade de Saúde Pública da USP; 2011.

c SABE is a multicenter study coordinated by the Department of Epidemiology of Faculdade de Saúde Pública, Universidade de São Paulo, conducted in seven cities in Latin America and the Caribbean. Available from: http://www.fsp.usp.br/sabe/

d Souza TBG. Religiosidade e Envelhecimento: panorama dos idosos do município de São Paulo. Estudo SABE [dissertation]. São Paulo (SP): Escola de Enfermagem da USP; 2011.
