# Supplementary material for: Religiousness and health-related quality of life of older adults
Source: Rev Saude Publica. 2015 Sep 10;49:55. doi: 10.1590/S0034-8910.201504900541 (PMC4544361; doi:10.1590/S0034-8910.201504900541)
Supplement: Translation

# Translation: Religiosidade e qualidade de vida relacionada à saúde do idoso

**Authors:** Gina Andrade Abdala, Miako Kimura, Yeda Aparecida de Oliveira Duarte, Maria Lúcia Lebrão, Bernardo dos Santos

**Affiliations:** I Programa de Pós-Graduação em Promoção da Saúde. Centro Universitário Adventista de São Paulo. São Paulo, SP, Brasil; II Escola de Enfermagem. Universidade de São Paulo. São Paulo, SP, Brasil; III Faculdade de Saúde Pública. Universidade de São Paulo. São Paulo, SP, Brasil

**Keywords:** Idoso, Nível de Saúde, Religião e Psicologia, Espiritualidade, Relações Interpessoais, Qualidade de Vida, Fatores Socioeconômicos, Inquéritos Epidemiológicos

## Abstract

**OBJETIVO:**

Analisar se a religiosidade exerce efeito mediador na relação entre fatores sociodemográficos, multimorbidade e qualidade de vida relacionada à saúde em idosos.

**MÉTODOS:**

Este estudo transversal, de base populacional, é parte do estudo Saúde, Bem-Estar e Envelhecimento (SABE). A amostra foi composta por 911 idosos da cidade de São Paulo, residentes na comunidade. A modelagem de equações estruturais foi realizada para verificar o efeito mediador da religiosidade na relação entre variáveis selecionadas e a qualidade de vida relacionada à saúde dos idosos, com modelos para homens e mulheres. As variáveis independentes foram: idade, escolaridade, funcionalidade familiar e multimorbidade. A variável de desfecho foi a qualidade de vida relacionada à saúde, medida pelo SF-12 (componentes físico e mental). As variáveis mediadoras foram a religiosidade organizacional, a não organizacional e a intrínseca. Os coeficientes alfa de Cronbach foram: componente físico = 0,85; componente mental = 0,80; religiosidade intrínseca = 0,89 e Apgar familiar = 0,91.

**RESULTADOS:**

Maiores níveis de religiosidade organizacional e intrínseca estiveram associados a melhor componente físico e mental para os idosos. Maior escolaridade, melhor funcionalidade familiar e menor número de doenças contribuíram diretamente para melhor desempenho nos componentes físico e mental, independente da religiosidade. Para as mulheres, a religiosidade organizacional mediou a relação entre a idade e os componentes físico (β = 2,401; p < 0,01) e mental (β = 1,663; p < 0,01). Para os homens, a religiosidade intrínseca mediou a relação entre a escolaridade e o componente mental (β = 7,158; p < 0,01).

**CONCLUSÕES:**

A religiosidade organizacional e intrínseca exercem efeito benéfico sobre a relação entre idade, escolaridade e a qualidade de vida relacionada à saúde desses idosos.

## INTRODUÇÃO

O envelhecimento populacional no Brasil vem ocorrendo de forma constante e acentuada. O percentual de idosos no município de São Paulo foi 12,5% em 2012, pouco maior que os percentuais do estado de São Paulo (12,1%) e do Brasil (11,3%).^6^ Segundo projeção para 2050, a proporção de idosos ultrapassará 22,7% da população total.^a^

É comum que pessoas idosas sejam portadoras de doenças crônicas não transmissíveis. Alterações físicas, emocionais e psicossociais decorrentes dessas doenças podem comprometer, em diferentes graus, a percepção de bem-estar e qualidade de vida (QV) dos idosos. Além disso, a multimorbidade pode interferir na realização das atividades instrumentais, sendo fator preditivo de pior qualidade de vida relacionada à saúde (QVRS) em idosos.^11^

Outros fatores que também podem explicar a QVRS de idosos são a idade, o sexo, a escolaridade e a funcionalidade familiar (Apgar familiar). Quanto maior a idade, pior é o desempenho nas dimensões da QVRS.^b^ O sexo feminino apresenta piores níveis tanto no componente físico quanto no mental.^20^ Quanto mais anos de escolaridade, melhor é a QVRS física e mental.^6^ ^,^ ^21^ Uma boa funcionalidade familiar também é preditora de melhor QVRS.^2^

O envelhecimento populacional aumenta cargas de doenças e incapacidades. Entretanto, a prevenção dessas doenças é efetiva mesmo na velhice, sendo, portanto, o principal foco para mudar o perfil atual de doenças crônicas.^23^

No estudo Saúde, Bem-Estar e Envelhecimento (SABE), de 2000,^c^ observou-se desigualdade no uso e acesso aos serviços de saúde e inadequação do modelo de atenção aos idosos, indicando necessidade de políticas públicas que levem em conta as especificidades dessa população.^13^

No planejamento de ações voltadas aos idosos, é necessário conhecer as condições de saúde e qualidade de vida dessa população, assim como os fatores que as determinam. Qualidade de vida é um conceito multidimensional e subjetivo. Na ausência de consenso conceitual, termos como satisfação com a vida, bem-estar, felicidade, boa vida, valor da vida e estado funcional têm sido usados.^9^ Já a QVRS é definida como o valor atribuído à duração da vida, modificado pelos prejuízos, estados funcionais, agravos à saúde, tratamentos ou políticas públicas, percepções e oportunidades sociais que são influenciados pela doença. A QVRS foi introduzida para estreitar o foco nos efeitos dos problemas de saúde e das intervenções terapêuticas sobre a qualidade de vida.^16^ Vários estudos sobre envelhecimento, saúde e qualidade de vida avaliam o efeito protetor das crenças e práticas religiosas/espirituais. Crescente número de estudos vem mostrando correlação positiva entre religião/espiritualidade e saúde física e mental.^3^ ^-^ ^6^ ^,^ ^8^ ^,^ ^14^ ^,^ ^15^

A importância da religião e sua prática relacionada com a QV foi positivamente avaliada por 50 indivíduos entre 65 e 86 anos.^18^ A religião foi considerada extremamente importante para a vida deles (p ≤ 0,05), dando força para suportar os problemas, perdas e lutas. Além disso, o contato com o divino é importante, pois traz segurança e dá conforto espiritual.^18^

Religiosidade é entendida como o grau em que o indivíduo acredita, segue e pratica uma religião. Compreende a dimensão organizacional, não organizacional e intrínseca. A organizacional está relacionada com a participação pública, em serviços religiosos nos templos ou igrejas. A não organizacional envolve a prática de atividades fora de uma instituição religiosa. A intrínseca refere-se a crenças, aspectos psicológicos da religião, conhecimentos e atitudes relacionadas à experiência religiosa.^11^

A religião pode afetar positivamente a saúde física e mental por meio de rede de apoio social, redução de comportamentos não saudáveis, redução da pressão arterial e tensão muscular durante oração e meditação e maior adesão a tratamentos médicos e cuidados preventivos.^1^

A religiosidade é uma característica marcante da população brasileira: 95,0% da população têm uma religião, 83,0% consideram-na muito importante em suas vidas e 37,0% participam de um serviço religioso ao menos uma vez por semana.^19^

Em estudo comparativo do perfil da religiosidade dos idosos de São Paulo, participantes do estudo SABE nos anos de 2000 e 2006, foi observado que ter uma religião e atribuir importância a ela impactaram positivamente a percepção de saúde.^d^

Em outra investigação, com base no mesmo estudo, foram analisados os fatores associados à QVRS. Entre diversos potenciais preditores, duas variáveis religiosas foram incluídas (filiação religiosa e importância da religião), mas nenhuma delas esteve associada com os componentes físico e mental do questionário Short Form-12.^b^

O objetivo deste estudo foi analisar se a religiosidade exerce efeito mediador na relação entre fatores sociodemográficos, doenças e qualidade de vida relacionada à saúde em idosos.

## MÉTODOS

Estudo observacional, de corte transversal, com abordagem quantitativa do estudo SABE. Foram estudados 911 idosos com 60 anos ou mais da zona urbana do município de São Paulo. Foram excluídos aqueles com declínio cognitivo e cujos questionários não foram respondidos.

As variáveis independentes foram idade, sexo, escolaridade, número de doenças e funcionalidade familiar (Apgar familiar). O Apgar familiar varia de zero (melhor) a 20 (pior).

As variáveis dependentes foram as dimensões dos componentes físico e mental da QVRS, avaliadas pelo SF-12. As variáveis mediadoras foram as dimensões organizacional, não organizacional e intrínseca da religiosidade.

A análise de dados foi feita por modelagem de equação estrutural (MEE), utilizando-se o Statistical Package for the Social Science (AMOS-SPSS, versão 22). A MEE é uma técnica multivariada que possibilita a análise simultânea de uma série de relações entre as variáveis medidas e os construtos latentes.^7^ Para se obter o efeito indireto de uma variável e o desfecho nos componentes, os valores obtidos são multiplicados.^7^

Utilizou-se o método de máxima verossimilhança, comumente empregado para a estimação de parâmetros em modelos de equações estruturais. O intervalo de confiança (IC) adotado foi de 90%. Os índices de ajuste utilizados foram a raiz do erro quadrático médio de aproximação (RMSEA); o índice de ajuste comparativo (CFI) e o índice de Tucker-Lewis (TLI).^7^

Quanto às medidas de confiabilidade dos questionários, foi calculado o coeficiente alfa de Cronbach. Para o CF da QVRS, o coeficiente foi 0,85; para o CM, 0,80; para a RI, 0,89; e para o Apgar familiar, 0,91.

Também foi realizado o teste *t* de Student para comparar as médias dos componentes físico e mental por sexo.

Pesquisa aprovada pelo Comitê de Ética e Pesquisa da Faculdade de Saúde Pública da Universidade de São Paulo e pelo Conselho Nacional de Ética em Pesquisa (CONEP – Processo 1.345 de 14/3/2006 para o ano de 2006).

## RESULTADOS

A amostra populacional apresentou percentual maior de mulheres (58,9%). A média de idade variou de 60 a 94 anos, sendo 71,8 (DP = 8,0) para mulheres e 72,3 (DP = 9,0) para os homens. Observou-se que os homens foram predominantes somente na faixa etária de 60 a 69 anos. Já as mulheres representaram percentual maior nas faixas de 70 a 79 anos e de 80 ou mais (p < 0,001).

Quanto à escolaridade, as mulheres apresentaram média de 3,8 anos de estudo (DP = 3,5) e os homens, de 4,5 (DP = 4,2). A proporção de mulheres analfabetas foi maior (18,9%) do que a de homens (11,7%) (p < 0,001).

Diferenças entre os sexos também foram observadas em relação ao Apgar familiar. As mulheres tiveram média de 2,4 (DP = 4,6) e os homens, 2,2 (DP = 4,0). Na categoria boa funcionalidade familiar, homens e mulheres apresentaram 90,6% e 89,2%, respectivamente (p < 0,001).

A média do número de doenças referidas pelas mulheres e pelos homens foi de 1,6 (DP = 1,2) e 1,3 (DP = 1,1), respectivamente. As doenças mais citadas entre os idosos foram: hipertensão arterial (64,7% das mulheres e 57,3% dos homens), diabetes (prevalência de 21,6% nas mulheres e 19,5% nos homens) e doenças osteoarticulares (41,6% das mulheres e 18,7% dos homens).

Quanto à religião, foi predominante a católica (66,2%), seguida da evangélica (22,3%) e da espírita/kardecista (5,4%). Menor percentual declarou ser protestante (1,0%), budista (0,6%) ou não possuir nenhuma religião (1,7%). Associando sexo e denominação religiosa, verificou-se que existem mais homens católicos (72,9%) do que mulheres (61,2%) e, consequentemente, mais mulheres evangélicas (26,3%) do que homens (16,6%) (p < 0,001).

Quanto às médias dos componentes físico e mental da QVRS segundo sexo, os homens apresentaram médias maiores nos dois componentes. No componente físico, a média foi 47,6 (DP = 7,9) nos homens contra 43,4 (DP = 10,3) nas mulheres; no mental, foi 55,0 (DP = 7,8) nos homens contra 53,7 (DP = 9,1) nas mulheres.

A Tabela 1 mostra os dados referentes às dimensões da religiosidade. As idosas frequentam mais os cultos religiosos, praticam mais as atividades religiosas particulares e dão mais importância para a religião (p < 0,001). Também são elas que mais consideram que a “religião dá forças para enfrentar e ajudar a entender as dificuldades da vida”. Além disso, para elas, a religião traz mais “sentido à vida”. Elas também se consideram mais religiosas que os homens (p < 0,001).

**Tabela 1:** Distribuição dos idosos segundo sexo e dimensões da religiosidade. São Paulo, SP, Brasil, 2006. (N = 911)

Dimensões da religiosidade	Feminino	Masculino	Total da categoria

	%	%	%
RO- Frequência com que vai à igreja			
Nunca	7,4	12,5	9,5
Várias vezes ao ano	20,1	36,4	26,8
1 ou 2 vezes por mês	15,7	12,0	14,2
Quase toda semana	25,1	18,6	22,4
Mais de 1 x por semana	31,7	20,4	27,1
RNO- Frequência com que reza ou pratica a religião			
Quase nunca ou nunca	2,2	6,9	4,1
Somente em ocasiões especiais	0,7	9,7	4,4
Várias vezes por semana	3,8	7,1	5,1
1 x ao dia	35,9	41,9	38,4
Várias vezes ao dia	57,5	34,4	48,0
RI- Importância da religião na vida			
Nada importante	1,7	5,0	3,1
Regular	3,0	6,7	4,5
Importante	95,2	88,3	92,4
RI- Quanto a religião dá forças para enfrentar as dificuldades			
Nada	1,7	6,6	3,7
Não muito	4,3	12,8	7,8
Muito	50,9	51,0	51,0
Completamente	43,1	29,6	37,5
RI- Quanto a religião ajuda entender as dificuldades			
Nada	2,1	8,3	4,7
Não muito	7,2	12,3	9,3
Muito	50,0	52,5	51,0
Completamente	40,7	26,9	35,0
RI- Religião dá sentido à vida			
Nunca	2,2	8,8	5,0
Às vezes	6,9	12,2	9,1
Muito	42,0	47,9	44,4
Totalmente	48,9	31,0	41,5
RI- Quão religioso se considera			
Nada	2,6	5,1	3,6
Não muito	6,0	11,0	8,1
Um pouco	27,9	40,9	33,2
Muito	63,5	43,1	55,1

Fonte: Estudo SABE.^c^

RO: religiosidade organizacional; RNO: religiosidade não organizacional; RI: religiosidade intrínseca

Na aplicação da modelagem de equações estruturais, a idade influenciou na religiosidade organizacional (β = -0,034; p < 0,01) e esta influenciou no componente físico das mulheres (β = 2,401; p < 0,01). A estimativa do modelo foi TLI = 0,613; CFI = 0,731; RMSEA = 0,08 (p ≤ 0,05; IC = 90%) (Figura 1).

**Figura 1:** Representação do modelo estrutural e de mensuração usando a modelagem de equações estruturais com as influências das variáveis sobre o componente físico para homens e mulheres idosos. São Paulo, SP, Brasil, 2006.

A religiosidade não exerceu efeito mediador entre os fatores sociodemográficos, doenças e o componente físico dos homens. Porém, maior escolaridade (β = 1,186; p = 0,04) e menor número de doenças (β = 8,108; p < 0,01) influenciaram diretamente para um melhor desempenho no componente físico dos homens, independentemente da religiosidade, mostrando que essas variáveis têm influência direta sobre a qualidade de vida relacionada à saúde dos idosos.

Para a melhoria do componente físico das mulheres, independentemente das dimensões da religiosidade, as variáveis que tiveram efeito foram: ter maior escolaridade (β = 1,055; p = 0,04), melhor funcionalidade familiar (β = 8,03; p < 0,01) e menor número de doenças (β = 9,61; p < 0,01).

Assim como no componente físico, a idade também esteve associada à religiosidade organizacional das mulheres (β = -0,034; p < 0,01) que, por sua vez, associou-se ao componente mental dessas mulheres (β = 1,661; p < 0,01). O modelo estimativo foi TLI = 0,613; CFI = 0,731; RMSEA = 0,08 (p ≤ 0,05; IC = 90%) (Figura 2). O efeito indireto total entre a idade, a religiosidade e o componente físico das idosas foi: idade → religiosidade organizacional → componente físico = -0,034 x 2,401 = -0,0813 (p ≤ 0,05), ou seja, para cada ano a mais da pessoa idosa, a religiosidade organizacional (ida à igreja) diminuía em 0,034; porém, esta aumentava em 2,401 o desempenho no componente físico da QVRS desses idosos.

**Figura 2:** Representação do modelo estrutural e de mensuração usando a modelagem de equações estruturais com as influências das variáveis sobre o componente mental para homens e mulheres idosos. São Paulo, SP, Brasil, 2006.

Analisando o efeito indireto total da relação entre a idade, religiosidade organizacional e componente mental das idosas, obteve-se o seguinte resultado: idade → RO → CM = -0,034 × 1,663 = -0,565. Pode se inferir que o efeito indireto da idade no componente mental, mediado pela religiosidade organizacional nas mulheres foi de -0,564. As dimensões do componente mental de maiores cargas foram a saúde mental (0,75) e a vitalidade (0,66) (Figura 3).

**Figura 3:** Síntese da modelagem de equações estruturais para o componente físico e mental, sexo feminino. São Paulo, SP, 2006.

Ao observar o modelo simples entre a escolaridade e o componente mental dos homens, mediado pela religiosidade intrínseca, encontrou-se o seguinte efeito total (direto + indireto): Escolaridade → religiosidade intrínseca → componente mental (-0,014 × 7,158) + (0,507) = -0,1002 + 0,507 = 0,4067 (Figura 4).

**Figura 4:** Síntese da modelagem de equações estruturais para o componente mental, sexo masculino. São Paulo, SP, 2006.

## DISCUSSÃO

Neste estudo, pôde-se observar que as idosas são mais religiosas do que os idosos em todas as dimensões da religiosidade, principalmente na frequência à igreja. Apesar das diferenças culturais de cada país e das educações religiosas que cada um recebe, usualmente, a literatura indica uma diferença entres os sexos, mostrando que as mulheres são mais abertas para expressar seus sentimentos religiosos, comentar, participar e se envolver nas tarefas da igreja. Elas também são mais íntimas com Deus na hora da morte, enquanto os homens são mais passivos e comentam menos seus sentimentos.^20^

Apesar de estarem com níveis mais elevados de religiosidade que os homens, as mulheres idosas apresentaram piores escores nos componentes físico e mental da QVRS. Em estudo com 1.942 idosas da Califórnia, USA,^22^ usando o SF-36, foi encontrado escore semelhante no componente físico (43,4; DP = 11,3) e escore um pouco maior para o componente mental (56,6; DP = 7,3). As mulheres também convivem mais com incapacidades físicas e pior qualidade de vida no domínio físico, principalmente as menos escolarizadas.^21^

Ao testar o modelo de como as variáveis sociodemográficas e de multimorbidade afetam a QVRS dos idosos, tendo a religiosidade como mediadora, observou-se que a idade foi um fator preditor negativo para o componente físico da QVRS das idosas. No entanto, a religiosidade organizacional funcionou como variável mediadora nessa relação, predizendo melhor qualidade de vida no componente físico das idosas. Nesta população, 49,5% dos idosos frequentam cultos religiosos quase toda semana, uma vez ou mais que uma vez por uma semana, sendo a maioria do sexo feminino.

A frequência à igreja promove apoio social e é atividade regular para muitos idosos.^6^ Em estudo com 426 idosos de uma comunidade do Canadá, observou-se que a espiritualidade não foi fator significante na melhoria da qualidade de vida (r = 0,19). Entretanto, os fatores preditores mais fortes para a qualidade de vida foram apoio social (*t *= 9,87; p < 0,001) e satisfação com a saúde (*t *= 6,854; p < 0,001), influenciados pelo convívio proporcionado pela adesão a uma comunidade religiosa.^17^

No presente estudo, a religiosidade apresentou relação mediadora com o componente físico apenas entre as idosas, possivelmente porque elas participam com maior frequência dos cultos religiosos, o que gera uma rede de suporte social e as ajuda a lidar com as doenças adversas.^6^

Maior escolaridade e menor número de doenças se comportaram como preditores diretos de melhor componente físico da QVRS em idosos de ambos os sexos. Esses dados corroboram os resultados de outros estudos.^6^ ^,^ ^11^ ^,^ ^21^

Ao observar o mesmo modelo em relação ao componente mental de QVRS, observou-se que a idade também funcionou como fator preditor negativo para esse componente nas idosas. No entanto, a religiosidade organizacional funcionou como variável mediadora nesta relação, predizendo melhor saúde mental.

Estudo com 499 idosos da Carolina do Norte, USA, investigou o envolvimento religioso público e fatores sociais da qualidade de vida, constatando que idosos com religiosidade mais profunda eram mais propensos a ver os amigos (62,0%), tinham melhor saúde referida (51,0%), menos sentimentos de depressão (63,0%) e achavam a vida mais entusiasmante (49,0%) em comparação com os menos religiosos. O estudo mostrou ainda que os idosos com deficiência física se beneficiavam mais da religiosidade, tanto relacionada à prática pública quanto à subjetiva, comparados àqueles sem deficiência física.^8^

Pertencer a uma religião e valorizá-la representa importante mecanismo de apoio para os idosos no enfrentamento de seus problemas diários, contribuindo para maior satisfação com a vida e menos sentimento de desamparo e desesperança.^5^

Outros estudos com idosos confirmam que, quanto maior o envolvimento religioso intrínseco, maior é a satisfação com a vida.^3^ ^,^ ^4^ Examinando a interação entre religião e espiritualidade em 277 idosos de uma coorte na cidade de Kansas, USA, encontrou-se que a religião não apresentou relação com a QVRS, mas a espiritualidade e a qualidade de vida estiveram associadas (p < 0,01) com o estado de saúde referido/saúde geral, mesmo ajustando para as covariáveis idade, raça, escolaridade e depressão. Daaleman et al^4^ relataram que a espiritualidade é um fator explicativo para o estado de saúde subjetivo em idosos.

No presente estudo, a escolaridade esteve inversamente relacionada à religiosidade intrínseca e esta positivamente relacionada ao componente mental dos homens, ou seja, a religiosidade intrínseca melhorava a relação da escolaridade com o componente mental deles.

A variável escolaridade (ter maior número de anos estudados) também influenciou diretamente para melhor desempenho do componente mental dos idosos neste estudo, em ambos os sexos. Em estudo realizado com 82 idosos de uma comunidade em Campinas, maiores níveis de escolaridade e renda estavam associados a uma melhor QV.^6^

Outros fatores que também apresentaram efeito direto no componente mental dos idosos neste estudo, foram o Apgar familiar (ter menor disfunção familiar) e menor número de doenças.

O papel da família está sendo considerado essencial na predição de melhor qualidade de vida nos idosos. Em um estudo com 210 pessoas idosas em Portugal, a qualidade de vida foi mais elevada entre aqueles com melhor funcionalidade familiar. Essa funcionalidade deve ser considerada no planejamento de ações de promoção da qualidade de vida para idosos.^2^

Quanto à multimorbidade, que pode interferir na locomoção dos idosos à igreja pela sua dificuldade para realizar esta atividade sozinho, também foi fator preditivo de pior QVRS. A multimorbidade funciona como variável supressora de QVRS, impedindo no seu bom desempenho.^11^

O delineamento deste estudo não permitiu estabelecer a relação causal, mas sim de preditor *versus* consequência, em que algumas variáveis sociodemográficas predizem uma melhor QVRS.

A escassa produção científica sobre a relação de variáveis sociodemográficas e de multimorbidade com a QVRS, mediada pela religiosidade e principalmente aplicando a MEE para a análise dos dados, prejudica a aproximação dos resultados para efeitos de comparação.

Os fatores ligados à QVRS de idosos suscitam atenção para as possíveis variáveis preditoras, que ajudarão a desvendar o melhor caminho na atenção à saúde física e mental da pessoa idosa, sugerindo mais pesquisas nesta área.

Muito embora religiosos ou profissionais da saúde possam debater sobre os benefícios ou riscos para os problemas mais sérios de saúde, pessoas que estão lutando com situações difíceis da vida, como multimorbidade, perdas e estresse podem usar sua crença e suas práticas religiosas para se readaptarem, pois elas representam um dos fatores mais importantes para o enfrentamento do estresse.^12^

Existem ao menos 20 escalas que ajudam a diagnosticar e direcionar assistência espiritual ao indivíduo. Dezoito delas foram validadas no Brasil e os autores sugerem que sejam usadas para identificação de diagnósticos com o objetivo de assistência de enfermagem numa perspectiva holística.^15^

Outra escala de espiritualidade recém adaptada e validada psicometricamente no Brasil é a *Daily Spiritual Experience Scale*, aplicada a 179 pacientes médico- cirúrgicos, mostrando confiabilidade e validade junto a pacientes hospitalizados.^10^

A análise estatística por meio da MEE foi útil para esclarecer os objetivos propostos e observar os efeitos diretos e indiretos das variáveis independentes em relação à QVRS dos idosos, mediados pela religiosidade.

Essa análise contribuiu para preencher as lacunas dos outros estudos encontrados. Portanto, as hipóteses foram comprovadas, mostrando estatisticamente que idosos com idade mais avançada, menor escolaridade, melhor funcionalidade familiar e menor número de doenças crônicas terão maior nível de religiosidade. Também ficou comprovado que maior nível de religiosidade organizacional e intrínseca são preditores de melhor qualidade de vida física e mental nos idosos.

Considerando o que foi encontrado na literatura sobre a importância da atenção integral aos idosos e os importantes achados deste estudo, recomenda-se que os profissionais da saúde estejam preparados para tal. Que tenham habilidade de comunicação e intervenções na área de religião e espiritualidade, pois a religiosidade pode ser usada como incremento da assistência à pessoa idosa, aumentando a qualidade de vida relacionada à saúde desse grupo.

Com o envelhecimento populacional, é necessário promover a saúde, diminuindo o número de doenças coexistentes, aumentar a funcionalidade familiar, incentivar o aumento no nível educacional e promover o efeito mediador da religiosidade na melhoria da QVRS.

## Footnotes

a Instituto Brasileiro de Geografia e Estatística. Comunicação Social 27 de Novembro de 2008. Brasília (DF); 2008 [citado 2012 jan 26]. Disponível em: http://www.ibge.gov.br/home/presidencia/noticias/noticia_visualiza.php?id_noticia=1272&id_pagina

b Ribeiro KT. Fatores associados à qualidade de vida relacionada à saúde de idosos residentes no município de São Paulo – Estudo SABE: Saúde, Bem-Estar e Envelhecimento [tese de doutorado]. São Paulo (SP): Faculdade de Saúde Pública da USP; 2011.

c O SABE é um estudo multicêntrico coordenado pelo Departamento de Epidemiologia da Faculdade de Saúde Pública da Universidade de São Paulo. Estudam idosos em sete cidades da América Latina e do Caribe. Disponível em http://www.fsp.usp.br/sabe/

d Souza TBG. Religiosidade e Envelhecimento: panorama dos idosos do município de São Paulo. Estudo SABE [dissertação]. São Paulo (SP): Escola de Enfermagem da USP; 2011.
